# Medicine

**Published:** 1905-12

**Authors:** J. R. Charles


### Progress of the Medical Sciences.

### MEDICINE.

**Paratyphoid Fever.**—Several papers [1] have recently appeared on this subject, written both from the clinical and pathological points of view. The opinion is held by several writers that the disease is more common than is generally supposed. The unity of typhoid fever has for some time been doubted by many observers, and it is possible that the discrepancy which exists between the clinical evidence and the results of Widal's reaction may be partly explained by the presence of paratyphoid fever. "According to the statistics of the Johns Hopkins Hospital, two per cent. of all cases of typhoid fever present a negative Widal, but only four per cent. of proven cases give no reaction with the bacillus typhosus." The disease may occur in sporadic or epidemic form. Occasionally a mixed infection of the typhoid and paratyphoid organisms is met with. Up to the present different clinical types corresponding with the different varieties of the paratyphoid bacilli have not been definitely identified, although some of the authors regard the more fulminating and septicæmic form as associated with Type B. The age of the patients attacked and the seasonal incidence of the disease are the same as in true typhoid. Men are more frequently attacked than women, the proportion being roughly three of the former to one of the latter. The onset is frequently more abrupt than in true typhoid, the symptoms at this stage including vomiting, anorexia, headache, pains in the back and limbs, epistaxis, irregular fever, and very occasionally a rigor. In a few cases enlargement of the cervical and inguinal glands have been met with. The tongue is furred, general *malaise* increases, and labial herpes is not infrequently seen. Bronchitis is common. The bowels may become constipated, or there may be diarrhœa, the stools being light yellow and offensive. Abdominal distension is not a prominent symptom, though it may occur. Rose spots appear about the 21st day from infection; they are similar in character and distribution to those seen in true enteric, but may be twice the size. Scarlatiniform rashes, dark macular spots and petechiæ are also mentioned. The spleen may be felt in 50 to 75 per cent. of the cases. Although hebetude may be marked, a hyperacuity of mind instead of the characteristic somnolence of enteric fever is noted

[1] H. Fox, *Univ. Penn. M. Bull.*, 1905, xviii. 52; A. B. Smallman, *J. Roy. Army Med. Corps*, 1905, v. 137; P. Mackie, *Lancet*, 1905, ii. 874; E. Cautley *Brit. J. Child. Dis.*, 1905, ii. 241; W. G. Savage, *J. Path. and Bacteriol.*, 1905, x. 341; Parsons, *Brit. M. J.*, 1905, i. 305; J. N. Henry, *Am. Med.*, 1905, ix. 613.

in several cases. The pulse resembles that of typhoid, being slow in proportion to the elevation of the temperature; dicrotism is met with early. Intestinal hemorrhage occurs in about five per cent. of the cases. It is rarely severe. No case of perforation has been recorded. The urine is usually febrile in character, contains albumen, and frequently many bacilli. Numerous casts are sometimes seen. The diazo reaction is found in about a third of the cases, and indicanuria is not uncommon. Examination of the cellular elements of the blood gives no means of distinguishing this disease from enteric fever. Leucopenia is met with, followed by a lymphocytosis (54 per cent.) and a relatively large number of eosinophiles (10 per cent.). In one case the eosinophiles went up from 10 per cent. during the acute stage to 28·6 per cent. during convalescence, and later to 35 per cent. The diurnal variations in the temperature are more marked than in enteric fever. Very exceptionally, cases have been met with in which there was no pyrexia. The course of the disease is, as a rule, like one of mild typhoid. It is more liable, however, to end by crisis. Relapses occur. Widal's reaction is, as a rule, negative, unless the case is complicated by true enteric. The exceptions to this statement are briefly mentioned later.

Among the complications may be mentioned bronchitis, broncho-pneumonia, pleurisy, endocarditis, femoral phlebitis, meningitis, peritonitis, cystitis, nephritis, pyelonephritis, orchitis, cholecystitis and lithiasis, osteomyelitis, myositis, and lastly typhoid. The complications of the Type B infection are said to be more numerous and more purulent. The mortality of the disease is estimated at about six per cent.

*Morbid Anatomy.*—As a rule there is a general blood infection, the organisms being recoverable from the peripheral circulation. The spleen, if enlarged, shows the same macroscopical and microscopical changes as in true typhoid. The mesenteric glands may or may not be enlarged. There is cloudy swelling of liver and kidneys. Patches of local necrosis are found in the liver. In the intestine there is an absence of the typical typhoidal ulceration of Peyer's patches. In some cases no ulceration has been found. It is therefore possible that these are the cases which in past years have been described as typhoid fever without intestinal ulceration. Intestinal ulcerations, when met with, are very variable in character. In some cases there have been numerous ulcers, which have closely resembled those of dysentery, and have differed entirely from those of true typhoid. In all cases there was practically no alteration in Peyer's patches or the solitary follicles. Wells and Scott [1] describe the ulcers met with by them as seated in the lower ileum quite superficially, rarely going through the muscular wall, many having the submucosa as a base. They were sharply outlined, frequently coalescing, and lacking any

[1] *J. Infect. Dis.*, January 2nd, 1905.

reactive inflammatory process in their margins or beyond them. They were, however, slightly undermined. Some think that this form of intestinal ulceration is associated with the paratyphoid bacillus, Type A, while that form of the disease which is unassociated with intestinal lesions, the morbid anatomy of which is rather that of a septicæmic process, is due to infection with the paratyphoid bacillus, Type B.

*Bacteriology*.—The so-called paratyphoid bacilli belong to a group of organisms which are intermediate in their characteristics between the bacillus typhosus and bacillus coli communis. Other important organisms in the same group are B. enteritidis of Gärtner and B. dysentericus.

Of the paratyphoid bacilli two types are described, Type A and Type B respectively. Morphologically these two are alike, but differ in minor cultural peculiarities and in their serum reactions. They are actively mobile; their flagella are said to be about the same in number but longer than those of B. typhosus. The organisms have been recovered from the blood, urine, fæces, rose spots, spleen (by puncture), pus of abscesses, and the vagina. According to Henry, infection with Type B is very much more common than infection with Type A, being found in 69 out of 84 cases. Geographically the organisms are very widely distributed. In India the disease is apparently fairly common, and Percival Mackie suggests that this may be due to an exaltation of the virulence of the organisms by their passage through the bodies of animals, such as the pig, hares, pea fowl, cattle and buffaloes, which there feed on human excrement. In the case of cows, their milk also may be infected.

Clinically the majority of the cases seem to have been diagnosed by the presence of the symptoms of apparent typhoid, and absence of Widal's reaction. The serum tests for the different organisms of this group show considerable complexity. Thus W. G. Savage in a recent case isolated two organisms of identical morphology and cultural characteristics, which were both paratyphoid B. organisms. Widal's reaction in the case was entirely negative. The two organisms differed widely in their reactions to the patient's serum, one being clumped by a dilution of 1 in 70,000, the other only being clumped by a dilution of 1 in 1,000. In a second case he separated an organism from the urine, which, after full consideration, he considered to be a true typhoid bacillus. No paratyphoid organisms could be found. This patient's serum gave repeated negative Widal's, but agglutinated the paratyphoid bacillus separated from Case I. in a dilution of 1 in 5,000. This he regards as conclusive evidence of paratyphoid infection. "For while we now know that the serum reactions in typhoid are not entirely specific, and that paratyphoid bacilli are often agglutinated in uncomplicated typhoid fever cases, yet never in such high dilutions."

According to Fox, if there is a repeatedly positive agglutination of Type A in a typhoid case and no reaction at all to B. typhosus, the case may be considered a Type A infection. But this does not hold good with Type B. The serum from a case of Type B infection will clump B. typhosus. Isolation of the causative bacillus from the blood, fæces, &c., must under these circumstances be attempted. If a Type B culture is obtained the question of mixed infection remains. Certain graded dilutions of the patient's serum, to which is added definite amounts of Type B culture in the first experiments and B. typhosus in the second set, may be injected into the peritoneal cavities of guinea pigs. The organisms rendered harmless by the least amount of serum may be considered as the type of germ giving rise to the attack in question. A further test is Castellani's. This "depends upon the fact that the agglutinating property or body of a serum may be exhausted for both organisms giving the positive results when it is saturated with the etiological organism, while saturation by the co-agglutinated organism will not decrease the limit of agglutination for the causative factor."

J. R. CHARLES.

